# Genomic and Phylogenetic Dissection of SARS‐CoV‐2 Transmission Networks in Healthcare Workers

**DOI:** 10.1155/ijm/6610060

**Published:** 2026-04-21

**Authors:** Irmak Güzel, Ayça Arzu Sayıner, Özgür Appak, Ayşe Banu Demir, Ahmet Naci Emecen, Gül Ergör, Belgin Ünal, Oya Özlem Eren-Kutsoylu, Kemal Can Tertemiz, Yusuf Hakan Abacıoğlu

**Affiliations:** ^1^ Department of Medical Microbiology, Turkish Republic Ministry of Health, Nusaybin State Hospital, Nusaybin, Turkey; ^2^ Department of Medical Microbiology, Faculty of Medicine, Dokuz Eylül University, Izmir, Turkey, deu.edu.tr; ^3^ Department of Medical Biology, Faculty of Medicine, Izmir University of Economics, Izmir, Turkey, ieu.edu.tr; ^4^ Department of Public Health, Division of Epidemiology, Faculty of Medicine, Dokuz Eylül University, Izmir, Turkey, deu.edu.tr; ^5^ Department of Infectious Diseases and Clinical Microbiology, Faculty of Medicine, Dokuz Eylül University, Izmir, Turkey, deu.edu.tr; ^6^ Department of Respiratory Diseases, Faculty of Medicine, Dokuz Eylül University, Izmir, Turkey, deu.edu.tr; ^7^ Department of Medical Microbiology, Faculty of Medicine, Izmir University of Economics, Izmir, Turkey, ieu.edu.tr

**Keywords:** healthcare workers, SARS-CoV-2, whole genome sequencing

## Abstract

Understanding how SARS‐CoV‐2 circulates among healthcare workers (HCWs) is critical for maintaining safe hospitals and protecting communities. We retrospectively examined 93 archived nasopharyngeal swabs collected from SARS‐CoV‐2 positive HCWs and their close contacts at a Turkish university hospital between March 2020 and November 2021. Viral RNA was confirmed by RT‐PCR and sequenced with the QIAseq DIRECT SARS‐CoV‐2 workflow on an Illumina platform; genomes were aligned to the Wuhan‐H1 reference and analyzed with maximum‐likelihood phylogenetics. Sixty‐three individuals formed 20 epidemiologically defined clusters (40 HCWs, 23 contacts; mean age ≈ 34 years), most experiencing only mild or moderate illness. Sequencing quality improved as RT‐PCR cycle threshold values fell, and nearly 98% of each genome was covered at 10 × depth. Fourteen viral lineages were detected, yet samples from the same cluster differed by no more than two single‐nucleotide changes, indicating recent person‐to‐person spread. Phylogenetic reconstruction suggested single‐source introduction in three‐quarters of clusters, with the remainder reflecting multiple introductions. Observational data linked transmission events to inconsistent use of personal protective equipment and inadequate physical distancing in break rooms, shared offices, and household settings. By combining genomic and field evidence, this study clarifies the paths by which SARS‐CoV‐2 moved through a healthcare workforce, highlights the value of whole‐genome sequencing for outbreak resolution, and reinforces the continuing need for rigorous infection‐control practices even when overall case severity appears low to prevent future clusters and maintain healthcare resilience.

## 1. Introduction

Infectious diseases remain a major global public health challenge in the twenty‐first century [1]. The COVID‐19 pandemic has highlighted the vulnerability of healthcare workers (HCWs), who are at increased risk of infection due to their close and prolonged contact with both symptomatic and asymptomatic cases [2, 3]. Protecting HCWs is essential not only for maintaining healthcare services but also for interrupting chains of transmission.

Whole‐genome sequencing (WGS) enhances traditional epidemiological approaches by enabling detailed analysis of transmission dynamics, cluster differentiation, and outbreak mapping [4, 5]. Beyond identifying circulating variants, WGS provides critical contextual information that allows the exclusion or confirmation of suspected transmission links, particularly when combined with detailed contact‐tracing data.

In Turkey, previous studies have documented SARS‐CoV‐2 seroprevalence, infection risk, and occupational determinants among HCWs, as well as the genomic and phylogenetic characteristics of circulating variants across different regions [6–8]. However, genomic investigations that systematically integrate detailed epidemiological (filiation) data with WGS to reconstruct transmission networks during outbreak settings, particularly within healthcare facilities and their linked contacts remain limited. Moreover, although the epidemiological landscape of SARS‐CoV‐2 has evolved substantially since 2020, understanding transmission dynamics during the early pandemic—when genetic diversity was low and infection rates were high—remains critical for interpreting the strengths and limitations of genomic surveillance in healthcare settings. Insights gained from this period are broadly applicable to other emerging respiratory pathogens with similar transmission characteristics.

In this study, we examined transmission clusters involving SARS‐CoV‐2 RNA–positive HCWs and their contacts in a university hospital using WGS and phylogenetic analysis, with the aim of informing infection prevention practices and future genomic surveillance approaches in healthcare facilities.

## 2. Methods

### 2.1. Study Design and Epidemiological Data Collection

HCWs who were found to be SARS‐CoV‐2 RNA positive between March 2020 and November 2021 were registered on a daily basis as part of the filiation studies carried out by the Department of Public Health and the Occupational Health and Safety Unit at Dokuz Eylül University Hospital. Contacts of the HCWs with a positive SARS‐CoV‐2 reverse transcription‐polymerase chain reaction (RT‐PCR) test 48 h before the onset of symptoms or 7 days before the positive PCR test if asymptomatic were defined as contacts in the study. Contacts were followed up for symptoms for 1 week and tested with SARS‐CoV‐2 RT‐PCR at the end of the period. Contacts were classified as high risk or medium risk in accordance with international contact‐tracing guidelines. High‐risk contact was defined as face‐to‐face exposure for at least 15 min at a distance of < 2 m or household contact. Medium‐risk contact was defined as exposure occurring with appropriate use of personal protective equipment [9, 10].

HCWs who had epidemiologically defined contact with each other (high or medium risk) who were working in the same unit, who had been recently infected (< 1 month), and their contacts who tested positive for SARS‐CoV‐2 RNA were grouped into epidemiological clusters. Clusters consisted of a minimum of 2 and a maximum of 11 individuals. Demographic characteristics of the study population were obtained from medical records.

### 2.2. Sample Size and Selection

Between March 2020 and November 2021, 72 clusters containing 271 cases were identified based on epidemiological data.

Cases diagnosed at an external center, cases with SARS‐CoV‐2 RT‐PCR Ct values > 28, and cases without archived nasopharyngeal swab samples were excluded.

After these exclusions, 133 nasopharyngeal swab samples from 39 clusters with appropriate sampling dates were retrieved from the archive stored at −80°C and included in the study.

There were a minimum of 3 and a maximum of 22 months between the time the selected samples were collected and the time they were analyzed for this study.

### 2.3. Nucleic Acid Isolation and SARS‐CoV‐2 RNA Detection

**FIGURE 1:**
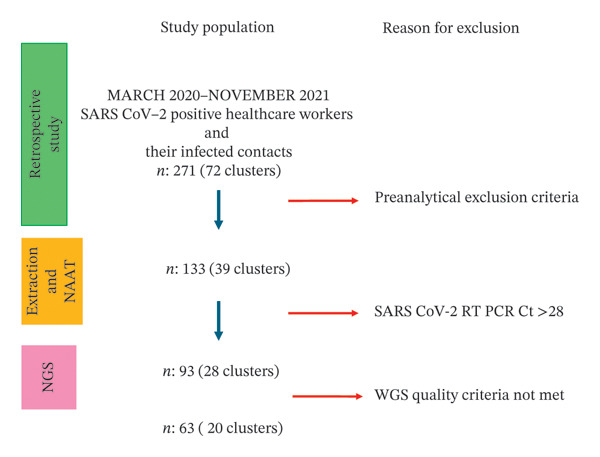
Flowchart illustrating the selection of the study population and reasons for sample exclusion. Between March 2020 and November 2021, epidemiological filiation data from 271 SARS‐CoV‐2–positive healthcare workers and their infected contacts, grouped into 72 clusters, were evaluated. Cases that did not meet preanalytical inclusion criteria were excluded, resulting in 133 samples from 39 clusters eligible for further analysis. Following nucleic acid extraction and confirmatory RT‐PCR testing, samples with insufficient viral load for sequencing were excluded, leaving 93 samples from 28 clusters for next‐generation sequencing (NGS). After whole‐genome sequencing and quality control, 63 high‐quality SARS‐CoV‐2 genomes representing 20 clusters met the predefined WGS quality criteria and were included in the final analyses. RT‐PCR: reverse transcription‐polymerase chain reaction; NGS: next‐generation sequencing; Ct: cycle threshold.

All archived nasopharyngeal swab samples underwent repeat RT‐qPCR testing. Nucleic acid extraction was performed using the EZ1 Virus Mini Kit v2.0 (QIAGEN) on the EZ1 Advanced XL, and SARS‐CoV‐2 RNA detection was carried out with the Realstar SARS‐CoV‐2 RT‐PCR Kit (Altona Diagnostics), targeting E and S genes. Samples with Ct ≤ 28 (*n* = 93, 28 clusters) were selected for WGS. Details of selected samples and clusters are shown in Figure 1 and Additional File 1.

### 2.4. WGS

Whole‐genome libraries were prepared with the QIAseq DIRECT SARS‐CoV‐2 Kit (QIAGEN). DNA was quantified (Qubit 4, HS‐dsDNA) and amplicon size verified (QIAxcel ScreenGel or 300–400‐bp agarose gel). Sequencing was completed in three runs: MiniSeq (33 samples) and two NextSeq 500/550 runs (60 samples); 13 samples were sequenced on both platforms. Illumina generated paired‐end 2 × 150 bp reads. Fastq files were mapped to the Wuhan‐H1 reference (MN908947.3) in CLC Genomics Workbench v22; unknown bases were masked as “N,” and consensus genomes > 28,500 bp were retained.

Of 93 datasets, 63 genomes (20 clusters) met quality criteria. Exclusions arose from the following:

a. 22 samples sequenced with MiniSeq had < 80% genome coverage, > 20% N bases, and < 90% 10 × depth;
b. three samples sequenced with NextSeq had < 95% genome coverage, > 7% N bases, and < 95% 10 × depth;
c. clusters with no remaining HCW samples after low‐quality sequences were removed.

### 2.5. Variation Analysis

The consensus sequence of each sample was compared to the Wuhan‐H1 sequence, and variations were determined on the CLC Genomics Workbench performed using the criteria of average quality score > 20 and frequency > 80% in accordance with the WHO guideline [11].

The characteristics of nucleotide variations and their effects on amino acid changes were evaluated in CLC Genomics Workbench and Nextclade web system. In addition, after multiple alignment of the sequences with MAFFT v7 [12], the variations compared to the reference were manually verified. The nucleotide numbering was started from the 5′UTR of the viral sequence, and the identified nucleotide and amino acid differences were indicated for each codon. Isolates in the same cluster were considered cluster‐associated if there were ≤ 2 single‐nucleotide variations (SNVs) in the whole‐genome analysis and cluster‐unassociated if there were > 2 SNVs or multiple‐nucleotide variations (MNVs).

### 2.6. Clade–Lineage Analysis

The FASTA files generated for each sample were uploaded to the Nextclade web system, lineages on Nextstrain, and clade characteristics on PANGOLIN were determined based on the combination of accumulated mutations. If different branches and/or lineages were identified in isolates in the same cluster, these isolates were considered unrelated to the cluster.

### 2.7. Phylogenetic Analysis

For the global phylogeny, the consensus sequences of 63 study samples were combined with 46 randomly selected external sequences from GISAID (https://www.gisaid.org/) and GenBank (https://www.ncbi.nlm.nih.gov/) and the reference Wuhan‐H1 sequence to create a dataset. In addition, for each defined clade, local clade‐specific datasets were created by randomly selecting external sequences reported from Turkey on similar dates together with the reference sequence. The most appropriate model for phylogenetic tree construction was determined using the “find the best DNA/Protein model” feature in MEGA 11.0 software. Maximum‐likelihood (ML) trees were constructed using the “GTR + G + I” model for the global tree and the “G + I” model for the clade trees with the lowest Bayesian information criterion (BIC) value and a bootstrap value of 200. Bootstrap support values ≥ 95% were defined as very strong, 95%–75% as strong, and < 75% as weak association. The tree was visualized by editing with FigTree (Version 1.4.4).

Weakly associated genome sequences that were initially reported in the same cluster but located in different regions on the tree were considered not associated with the cluster.

### 2.8. Statistical Analysis

Data were analyzed using SPSS 24.0 software. The suitability of the numerical data for normal distribution was evaluated by Kolmogorov–Smirnov tests. Descriptive statistics of numerical data were expressed as mean and standard deviation, and descriptive statistics of categorical variables were expressed as number and percentage. The Mann–Whitney *U* test was used for the comparison of numerical variables between two groups. The results were evaluated within a 95% confidence interval, and *p* < 0.05 values were considered statistically significant.

In the correlation of numerical variables, those that did not meet the normal distribution criteria were analyzed by the Spearman correlation test. Correlation coefficient (*r*) threshold values were evaluated as (+) positive, (−) negative, and 0.75–1.00: very strong, 0.50–0.74: strong, 0.25–0.49: moderate, and 0.24 and below: weak.

### 2.9. Ethics Approval and Consent to Participate

This study was conducted in compliance with the principles outlined in the Declaration of Helsinki. Ethical approval was obtained from the Dokuz Eylül University Clinical Research Ethics Committee (date: 11 May, 2020, No.: 2020/08‐28), along with authorization from the Turkish Ministry of Health, General Directorate of Health Services. Before participation, all participants were fully informed, both verbally and in writing, about the study’s purpose, scope, and procedures. Participants were assured of their right to withdraw from the study at any time, regardless of the outcomes, and their written informed consent was obtained on a voluntary basis.

## 3. Results

In the study, 93 samples in 28 clusters consisting of HCWs with SARS‐CoV‐2 RNA detected in DEU Hospital between March 2020 and November 2021, and infected contacts supported by filiation data were analyzed by the WGS method.

Thirty samples were excluded due to low sequence quality for reasons described in the Methods section. Sixty‐three samples in 20 clusters with high‐quality sequences were evaluated for further analyses. Data are shown in Additional File 2.

### 3.1. Epidemiological, Demographic, and Clinical Features

The study group consisted of 63 individuals, 40 were HCWs (mean age: 34.1 ± 9.3 years), and 23 were family members (mean age: 33.7 ± 21.3 years). The female rate was 56% (35/63). The units and contacts of infected HCWs are shown in Table 1. When the contact status was analyzed, HCWs were defined as medium‐ and high‐risk contacts, especially if they shared common working areas. Eating and drinking activities, changing rooms, social activities, and contact within the family were defined as high‐risk. There was no history of traveling abroad or contact with people from abroad among the HCWs and their contacts.

**TABLE 1:** Distribution of units and contacts of infected healthcare workers.

Contact	No. of clusters	
	Clinic	Nonclinic
Healthcare worker	7	3
Healthcare worker and family member	1	1
Family member	5	3
Total no.	63 persons in 20 clusters	

HCWs had the disease with no symptoms (*n*: 1) or with mild‐to‐moderate symptoms (*n*: 39). Symptoms included weakness, fatigue, mild fever, muscle pain, headache, sore throat, nasal congestion, cough, loss of taste, and smell. None of them required hospitalization or intensive care.

### 3.2. Relationship Between Cycle Threshold (Ct) Value and Sequencing Quality of Samples

**FIGURE 2:**
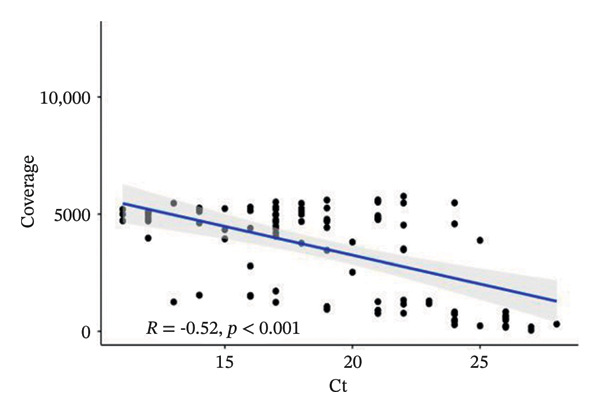
Spearman’s correlation between RT‐PCR Ct values and average coverage in the study samples. The scatter plot displays the negative correlation between Ct values (*x* axis) and coverage (*y* axis). The fitted regression line (blue) shows a significant inverse correlation, with a Spearman correlation coefficient of *R* = −0.52 and a *p* value < 0.001, indicating a statistically significant relationship (*R* = −0.52; *p* < 0.001) (Ct: cycle threshold).

Coverage (10 ×) and reference genome coverage results were evaluated in the quality filtering of WGSs from 93 samples. The mean Ct value was 24.5 (95% CI 22.6‐26.4) in samples with < 90% genome coverage and 17.9 (95% CI 17.1–18.8) in those with ≥ 90% (*p* < 0.001). Similarly, the mean Ct value was 24.2 (95% CI 22.2–26.3) in samples with reference genome coverage < 80% and 18.3 (95% CI 17.4–19.2) in those with ≥ 80% coverage (*p* < 0.001). Mean coverage values were strongly inversely correlated with Ct values, indicating that the higher the viral load, the better the genome coverage is (Figure 2).

In the 63 sequences that met the quality criteria, the total number of reads ranged from 820,000 to 5 million and the average consensus sequence length was 29,771 ± 77.3 bases. Average coverage was 4335 ± 1312 for sequences covering 82%–99% of the reference genome. At 10 × depth, the sequences covered an average of 97.9 ± 1.9% of the genome.

### 3.3. Clade and Lineage Classification of Sequences

**FIGURE 3:**
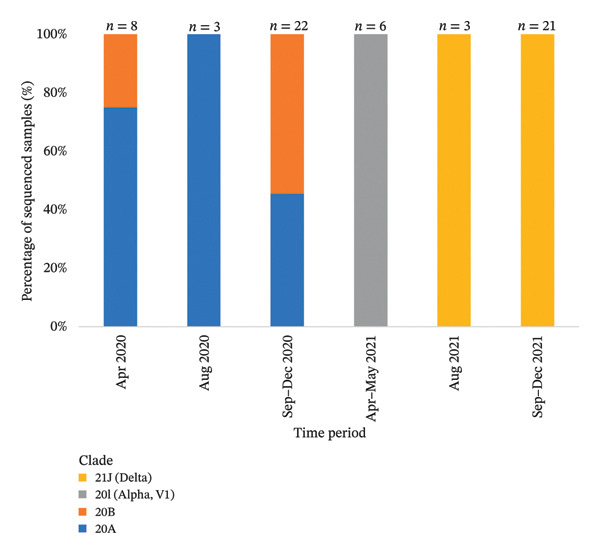
Bars represent the relative distribution (%) of SARS‐CoV‐2 clades within the sequenced dataset of this study, normalized to the total number of samples sequenced in each time interval (*n* shown above bars). Time intervals were defined based on sample availability and key epidemiological phases rather than uniform calendar periods.

**FIGURE 4:**
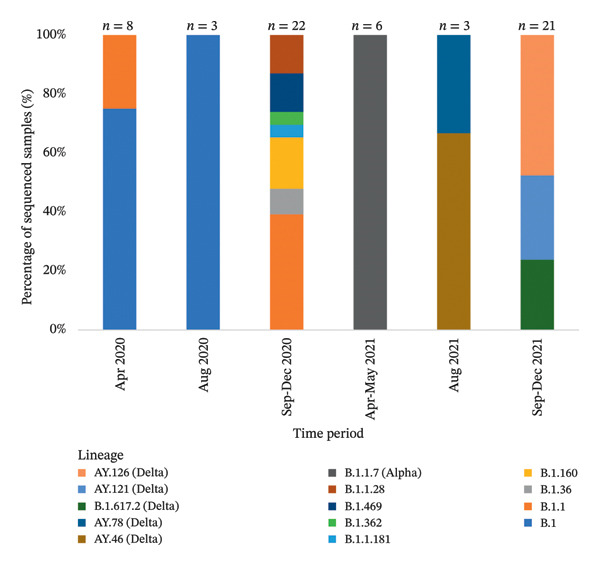
Bars represent the relative distribution (%) of SARS‐CoV‐2 lineages within the sequenced dataset, normalized to the total number of samples sequenced in each time interval (*n* shown above bars). Time intervals were defined based on sample availability and key epidemiological phases rather than uniform calendar periods.

Viral isolates included in the analyzed clusters were classified into 14 distinct SARS‐CoV‐2 lineages belonging to four major clades. To describe the temporal distribution of these clades and lineages within the study dataset, samples were grouped into time intervals defined according to the timing and availability of epidemiologically linked cases and sequencing activity, rather than uniform calendar periods. The normalized temporal distribution of clades and lineages across these epidemiologically defined intervals is presented in Figures 3 and 4.

### 3.4. Analysis of Nucleotide and Amino Acid Changes in Sequences

Compared to the reference Wuhan‐H1 sequence, 284 nucleotide variations were found in the studied viral isolates. Of these, 180 were nonsynonymous, 97 were synonymous, and 7 were noncoding region variants. SNVs were the most common (*n*: 275). There were four MNVs and seven deletions. A cytosine to thymine (C > T) conversion pattern was predominant in the variations (*n*: 120).

Of these variations, 55% (*n*: 157) were found in the ORF1ab region, which occupies two‐thirds of the viral genome. This was followed by the S (*n*: 52) and N (*n*: 28) gene regions.

Regarding nonsynonymous mutations (*n*: 180), 3 to 38 amino acid (aa) changes were detected per isolate. These changes were observed in ORF1ab (*n*: 89), S (*n*: 33), N (*n*: 19), and other gene regions in parallel with nucleotide changes. Details of MNVs and deletions are shown in Tables 2 and 3. All nucleotide variations and corresponding aa changes are shown in Additional File 3

**TABLE 2:** Multiple‐nucleotide variants (MNVs) identified in SARS‐CoV‐2 genomes.

Region	Type	Reference	Allele	Gene	AA substitution	Clade	No. of samples
28881…28883	MNV	GGG	AAC	N	R203K, G204R	20B + 20I	20
28280…28282	MNV	GAT	CTA	N	D3L	20I	6
27876…27877	MNV	TG	GT	ORF7b	C41V	20I	3
25970…25971	MNV	GG	TA	ORF3a	W193L	20A	1

Abbreviations: AA, Amino acid; MNV, multiple‐nucleotide variant.

**TABLE 3:** Deletion variants identified in SARS‐CoV‐2 genomes.

Region	Type	Reference	Allele	Gene	AA substitution	Clade	No. of samples
28271	Deletion	A	—	N‐ORF8	—	21J + 20I	30
22029…22034	Deletion	AGTTCA	—	S	E158G	21J	24
28248…28253	Deletion	GATTTC	—	ORF8	D119‐, F120‐	21J	24
11288…11296	Deletion	TCTGGTTTT	—	ORF1ab	S3675‐, G3676‐, F3677‐	20I	6
21765…21770	Deletion	TACATG	—	S	H69‐, V70‐	20I	6
21991…21993	Deletion	TTA	—	S	Y144‐	20I	6
27692…27697	Deletion	TTTTTC	—	ORF7a	F101‐, L102‐	21J	3

Abbreviation: AA, amino acid.

### 3.5. Phylogenetic Analysis

**FIGURE 5:**
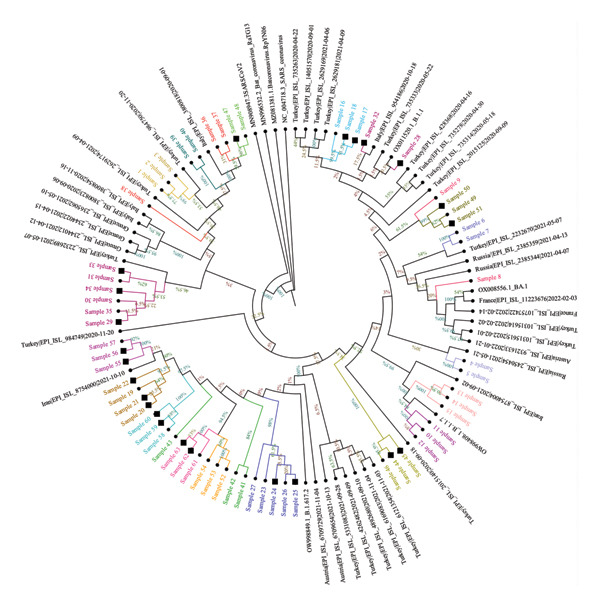
The maximum‐likelihood phylogenetic tree illustrates the relationships between global SARS‐CoV‐2 genomes. Color‐coding indicates the presumptive epidemiological clusters based on filiation data, and household contacts are represented by a square symbol. Sequences sourced from GenBank and GISAID are shown in black. The tree was constructed using sequences randomly selected from a similar timeframe globally, ensuring a comprehensive comparison. Bootstrap support values, displayed as percentages at the branch nodes, indicate the statistical reliability of each grouping. The scale bar represents the genetic distance, with branch lengths corresponding to the degree of sequence divergence.

Phylogenetic trees were constructed using the ML method with datasets prepared with consensus sequences of 63 samples, the reference strain, and external sequences obtained from GISAID and GenBank. The analysis with global external sequences is shown in Figure 5, and the analysis with clade‐specific sequences from Turkey is shown in Figure 6.

FIGURE 6Phylogenetic trees illustrating the relationships between SARS‐CoV‐2 sequences within the (a) 20A, (b) 20B, (c) alpha, and (d) delta clades. Different colors are used to denote the epidemiologically defined sample clusters based on filiation data, while square symbols indicate the household contacts. Sequences obtained from GenBank and GISAID databases are indicated in black, distinguishing them from the local sequences analyzed in this study. The trees were constructed using sequences from Turkey, all collected within a similar timeframe, ensuring a more accurate representation of the evolutionary relationships specific to the region. Bootstrap support values, displayed as percentages at the branch nodes, indicate the statistical reliability of each grouping. The scale bars represent genetic distance, with branch lengths corresponding to the degree of sequence divergence.

**Figure:**
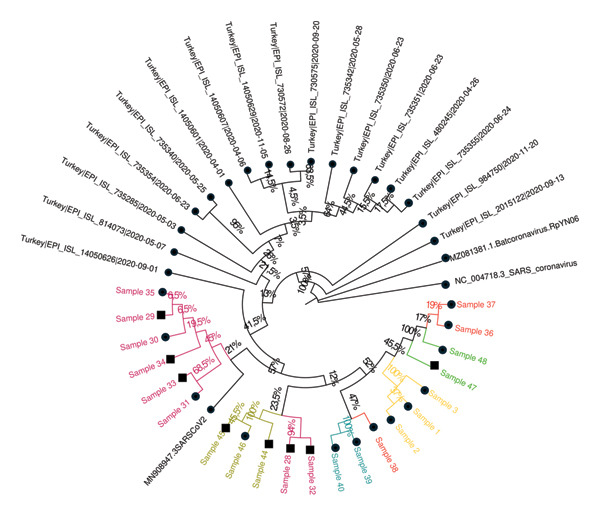
(a)

**Figure:**
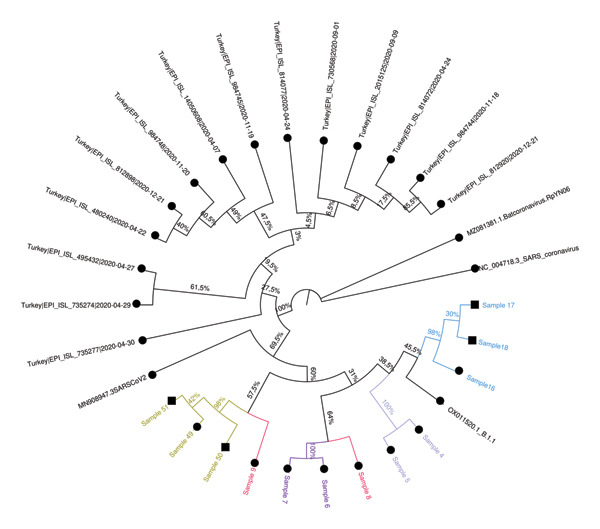
(b)

**Figure:**
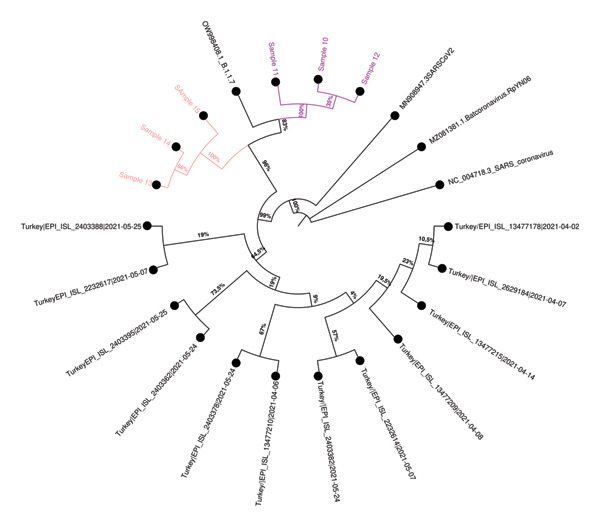
(c)

**Figure:**
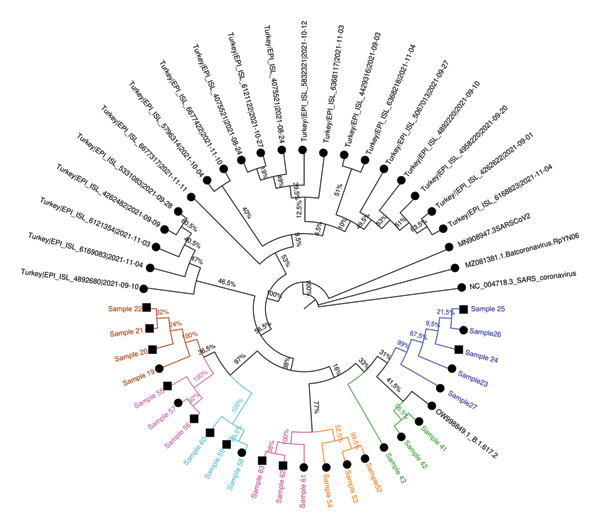
(d)

### 3.6. Cluster Analysis Results

Among the 20 epidemiologically defined clusters evaluated by WGS, 15 clusters (Clusters 1–3, 5–9, 12, 14–16, and 18–20) showed strong concordance between epidemiological and molecular findings. In these clusters, most individuals shared identical nucleotide variations or differed by no more than two SNVs and formed well‐supported groupings in the ML phylogenetic tree (bootstrap support > 95%). These concordant clusters predominantly comprised either HCWs with documented close workplace contact or HCWs linked to small household units, supporting transmission patterns consistent with filiation‐based exposure histories.

In contrast, five clusters (Clusters 4, 10, 11, 13, and 17) demonstrated marked discordance between epidemiological and genomic findings, underscoring limitations of filiation‐based cluster definitions when used in isolation. Four of these discordant clusters (Clusters 4, 11, 13, and 17) consisted exclusively of HCWs, whereas Cluster 10 represented a larger household‐based cluster involving nine individuals residing in the same apartment building, including four HCWs.

Detailed assessment of these discordant clusters revealed heterogeneous patterns of inconsistency. In Cluster 4, which included two individuals, sequences differed by more than two SNVs and were assigned to distinct lineages with clearly separated phylogenetic positions, excluding a direct transmission link despite epidemiological association. Cluster 10 showed a more complex structure: Two samples displayed MNV differences and belonged to different clades and lineages, while the remaining individuals exhibited only weak phylogenetic clustering, providing limited genomic support for a single transmission chain within this extended household setting. Similarly, in Clusters 11, 13, and 17, at least one individual per cluster differed from the remaining members by more than two SNVs and was assigned to a distinct lineage, with weak or inconsistent phylogenetic placement. Together, these findings indicate that some epidemiologically inferred links—particularly within clusters characterized by more complex contact patterns or heterogeneous exposure histories—were not supported by genomic evidence.

A comprehensive table presenting the results of WGS analyses, clade–lineage distributions, and phylogenetic analyses for samples from the 20 clusters is provided in Additional File 4.

## 4. Discussion

HCWs represent a critical interface between the hospital and the community and were therefore a key population in the transmission of SARS‐CoV‐2 during the COVID‐19 pandemic. In this study, we combined WGS with detailed epidemiological filiation data to investigate transmission networks among infected HCWs and their contacts. This integrated approach allowed us to compare phylogenetic relationships with reported exposure histories and to evaluate the strengths and limitations of epidemiology‐based cluster definitions. Our primary finding was that genomic data both confirmed and refuted suspected transmission links within epidemiologically defined clusters. Of the 20 clusters analyzed, 15 showed strong concordance between two types of data, with viral genomes differing by no more than two SNVs and forming well‐supported phylogenetic groupings. In contrast, five clusters displayed clear discordance between filiation data and genomic evidence, indicating independent acquisition events rather than direct intracluster transmission. These results suggest that epidemiological clustering alone can lead to an overestimation of healthcare‐associated transmission, particularly in settings with intense community spread and multiple exposure opportunities for HCWs.

The evolutionary rate of SARS‐CoV‐2 has been estimated at approximately two to three nucleotide substitutions per month, particularly during the early pandemic period when genetic diversity was limited. On this basis, differences of 0–2 single‐nucleotide polymorphisms (SNPs) have been widely used as a threshold for identifying recent transmission events. In our study, clusters that exceeded this threshold or that contained MNVs or lineage discordance, were unlikely to represent direct transmission chains. These observations are consistent with previous reports showing that WGS may be more effective in excluding suspected transmission than in definitively confirming it during periods of high transmission and low genetic diversity.

In order to interpret transmission patterns inferred from genomic data, it is essential to understand viral evolutionary dynamics. The evolutionary rate of the circulating SARS‐CoV‐2 lineage can be used to determine the probability of the virus accumulating mutations over the course of transmission. Since 2020, studies have estimated the evolutionary rate of the Wuhan strain and subsequent variants at approximately two to three nucleotide substitutions per month and 24–34 substitutions per year [13, 14]. On this basis, SNP–based analyses have been particularly informative in resolving hospital‐associated infections, and a difference of 0–2 SNPs has been widely interpreted as indicative of linked transmission occurring within the preceding days or weeks [15–17].

In our study, viral isolates from infected HCWs and their contacts across 20 epidemiologically defined clusters were analyzed using WGS. In five clusters, the presence of MNVs (> 2 SNPs or MNVs) was more consistent with independent acquisition events than with direct intracluster transmission. Re‐evaluation of epidemiological data indicated that HCWs showing discordance between epidemiological clustering and phylogenetic patterns frequently reported moderate‐risk contact with cluster members, as well as multiple exposures to infected patients who could not be included in the analysis. While several samples grouped together within the same epidemiological clusters, others exhibiting multiple‐nucleotide polymorphisms were assigned to different viral clades or lineages and were positioned distantly on the phylogenetic tree. Together, this finding supports the likelihood that infection sources in these cases were external to the defined clusters rather than reflecting direct transmission among cluster members. An important exception was observed in two clusters (Clusters 11 and 15). In these clusters, HCWs infected over a period of approximately 3 weeks shared identical cluster‐defining mutations and were positioned in close proximity on the phylogenetic tree. This pattern suggests a potential shared exposure or overlapping transmission context that may not have been fully captured by routine epidemiological investigation alone.

In line with these observations, epidemiological data combined with next‐generation sequencing have been used throughout the COVID‐19 pandemic to investigate suspected in‐hospital transmission clusters [18, 19]. However, our findings indicate that during periods of limited viral genetic diversity and intense community transmission, genomic surveillance may have limitations in resolving SARS‐CoV‐2 transmission events. Similar challenges have been reported in previous studies. Meredith et al. showed that 22% of genetic clusters with no SNP differences lacked identifiable epidemiological links [17]. Likewise, Braun et al. reported that early in the outbreak WGS may be more informative for excluding transmission than confirming it [20].

Despite these limitations, the integration of robust epidemiological data with phylogenetic analyses in our study helped mitigate some of the challenges related to low viral genetic diversity. Clusters supported by strong epidemiological links showed concordant placement within phylogenetic trees constructed using sequences from dominant clades circulating in Turkey during similar time periods. This approach reduced the risk of misclassification resulting from coincidental genetic similarity.

In contrast to previous national studies that have largely relied on descriptive or population‐level genetic analyses, our study combines detailed epidemiological (filiation) data with WGS to investigate transmission dynamics within defined outbreak clusters among HCWs in a university hospital setting. This integrated framework provides additional contextual depth for interpreting transmission patterns involving HCWs within the hospital‐related environment.

Beyond methodological considerations, identifying potential sources of exposure remains critical for interpreting transmission among HCWs. Previous studies have shown that in approximately 40% of in‐hospital outbreaks, either the primary case could not be identified or was not a patient with COVID‐19 [21]. These findings highlight the substantial contribution of community transmission, often described as reverse transmission, to the dissemination of SARS‐CoV‐2.

Household exposure has been repeatedly identified as an important source of infection [22, 23]. Consistent with these findings, several clusters in our study included HCWs and household members who shared similar mutations and close phylogenetic proximity, supporting domestic exposure as a relevant transmission pathway. These observations underscore the importance of targeted monitoring and early isolation of HCWs following household exposure. Implementation of preventive measures within the household, including appropriate mask use, disinfection practices, and, where feasible, provision of temporary accommodation for HCWs, has been shown to effectively reduce secondary transmission [24]. Our findings reinforce the relevance of these interventions in mitigating infection risk among HCWs.

In addition to household exposure, transmission between coworkers represents another major risk of infection for HCWs [25, 26]. In our study, high‐risk exposures were frequently associated with social and shared areas within the hospital, including eating and drinking spaces, changing rooms, and other common areas. Inconsistent use of personal protective equipment and inadequate adherence to physical distancing in these settings likely contributed to transmission events. Several factors may underlie these behaviors, including suboptimal physical conditions, insufficient awareness, and occupational burnout. Consistent with our observations, previous studies have identified poor compliance with infection prevention measures—particularly personal protective equipment—as a significant contributor to SARS‐CoV‐2 transmission among HCWs [27]. Although higher seroprevalence has been reported among nonclinical HCWs in some settings [28, 29], infected HCWs in our cohort were predominantly from clinical departments, underscoring the importance of continuous reinforcement of infection prevention and control (IPC) practices across all staff groups. Education and awareness regarding transmission risks among HCWs therefore remain essential components of infection prevention. In‐depth assessments of HCWs’ risk perception, high‐risk contact scenarios, and adherence to IPC measures may provide valuable insights for targeted interventions and behavioral modification strategies, particularly in high‐risk occupational settings.

In addition to behavioral and occupational factors, individual clinical characteristics of HCWs also influence transmission dynamics and detection strategies. In this study, the mean age of the infected HCWs was 34.1 ± 9.3 years, and the majority reported mild‐to‐moderate symptoms. A meta‐analysis by Sahu et al. demonstrated that the incidence of severe or critical illness is significantly lower in HCWs than in the general population, a finding attributed to younger age, fewer underlying conditions, and greater medical awareness [30]. Several studies have further emphasized that asymptomatic infections are common in HCWs [31, 32]. Therefore, reliance on symptom‐based surveillance alone may fail to detect a substantial proportion of potential transmission events in this population.

From a diagnostic and analytical perspective, ensuring high‐quality genomic data was a critical component of this study. Most low‐quality sequences (22/30) were generated using the MiniSeq platform. Following a direct comparison of MiniSeq and NextSeq performance on 13 samples, the NextSeq platform was used for the remainder of the study, as it consistently produced higher genomic coverage and a lower proportion of unknown bases. In addition, RT‐PCR Ct values, reflecting viral load in the sample, showed a strong inverse correlation with average sequencing coverage (*r* = ‐0.52; *p* < 0.001). Mean Ct values were significantly lower in samples achieving ≥ 90% genome coverage at 10 × depth and ≥ 80% reference genome coverage (*p* < 0.001). Together with recent evidence, our findings support the use of Ct values as a practical preanalytical screening criterion for WGS, as lower Ct values are consistently associated with higher genome coverage, successful whole‐genome assembly, and reliable lineage assignment, whereas higher Ct values are more likely to yield incomplete genomic data [33, 34].

Within this quality‐controlled dataset, WGS data of the samples revealed substantial viral genomic diversity across the study period. Fourteen different lineages belonging to four different clades of SARS‐CoV‐2 were identified, consistent with the dominant variants circulating in Turkey at the corresponding time points [35]. Overall mutation patterns were consistent with previous reports, including a predominance of C > T substitutions. Notably, no nucleotide changes were detected in the E gene, consistent with its characterization as a highly conserved region of the SARS‐CoV‐2 genome. Mutations were most frequently detected in the ORF1ab, spike, and N gene regions. However, this distribution likely reflects differences in the gene length and sequencing target size rather than true differences in the mutation density or selective pressure [36–39]. Overall, the mutation patterns observed in this study are consistent with previously reported genomic changes associated with viral fitness, structural stability, neutralization properties, and host immune interactions in SARS‐CoV‐2 [40–44]. Detailed mutation data for structural and nonstructural genes are provided in Additional File 3. Although the variants analyzed in this study are no longer predominant, the mutation patterns observed remain informative for understanding viral evolution during periods of intense transmission and limited genetic diversity. Importantly, the integrative genomic–epidemiological framework applied here remains relevant for the interpretation of genomic data from future emerging variants and other respiratory pathogens.

The study had some limitations. Transmission networks in some clusters could not be fully resolved because certain samples were excluded based on predefined criteria or yielded low‐quality genomic sequences. In addition, patients could not be included in the study due to a high number of potential contacts and the lack of sufficient epidemiological data. Identifying asymptomatic HCWs was also challenging except in cases where individuals reported known exposure to an infected colleague. Furthermore, although strong epidemiological and genomic associations were observed among cases, the findings should be interpreted as suggestive rather than confirmatory with respect to transmission direction. Temporal information, including symptom onset and PCR positivity dates, was used to support epidemiological inference. However, more definitive reconstruction of transmission direction would benefit from additional analytical approaches, such as time‐scaled phylogenetic analyses or estimation of the time to the most recent common ancestor (tMRCA), which could be incorporated in future studies. Despite these methodological considerations, the integrated use of genomic and epidemiological data in this study provides valuable insights into transmission dynamics among HCWs.

In conclusion, this study demonstrates that a comprehensive approach combining viral WGS with detailed epidemiological data meaningfully supports evidence‐based IPC strategies. Such integration can help limit transmission within healthcare settings while avoiding unnecessary quarantine measures or resource‐intensive interventions by accurately excluding unsupported transmission links.

**Nomenclature**
HCWs: Healthcare workers
WGS: Whole‐genome sequencing
RT‐PCR: Reverse transcription‐polymerase chain reaction
SNV: Single‐nucleotide variations
MNV: Multiple‐nucleotide variations
ML: Maximum likelihood
Ct: Cycle threshold
SNP: Single‐nucleotide polymorphism
NGS: Next‐generation sequencing
IPC: Infection prevention and control
PPE: Personal protective equipment
tMRCA: Time to the most recent common ancestor

## Author Contributions

Irmak Güzel: study implementation, data curation, formal analysis, conceptualization, visualization, and writing–original draft, review, and editing; Özgür Appak: data curation, conceptualization, and review and editing; Ayşe Banu Demir and Gül Ergör: study conception and design, study implementation, data curation, methodology, formal analysis, and conceptualization; Ahmet Naci Emecen: data curation, methodology, formal analysis, and conceptualization; Belgin Ünal, Oya Özlem Eren‐Kutsoylu, and Kemal Can Tertemiz: study conception and design, data curation, and methodology; Yusuf Hakan Abacıoğlu: study conception and design, data curation, methodology, conceptualization, and project administration; Ayça Arzu Sayıner: study conception and design, data curation, methodology, conceptualization, project administration, and review and editing.

## Funding

This work was supported by the Dokuz Eylül University Department of Scientific Research Projects (DEU‐BAP) (Project ID: 2461; Project Code: 2020.KB.MLT.008).

## Disclosure

All authors have read, revised, and approved the final manuscript. The funder had no role in study design, data collection and analysis, manuscript preparation, or the decision to publish.

## Conflicts of Interest

The authors declare no conflicts of interest.

## Supporting Information

Additional supporting information can be found online in the Supporting Information section.

The following supporting files provide additional data supporting the findings of this study:

All supporting materials are provided as final versions and have not been modified by the journal’s production team. Authors are responsible for their content.

## Supporting information


**Supporting Information 1** Additional File 1. A table presenting the detailed demographic characteristics and epidemiological cluster assignments of SARS‐CoV‐2–positive individuals included in the study. Cluster numbers reflect groupings based on filiation data, and information includes sample number, sex, age, and participant status.

**Supporting Information 2** Additional File 2. A comprehensive table of sequence analysis results from 63 individuals across 20 clusters. It includes demographic data, RT‐PCR Ct values, total reads, consensus length, unknown base percentages, average coverage, 10 × coverage, and reference coverage. It also contains clade and lineage classifications and metadata for sequences submitted to GISAID.

**Supporting Information 3** Additional File 3. A table summarizing all nucleotide variations identified in the sequenced SARS‐CoV‐2 genomes. Each entry includes the gene, region, type of variation, amino acid substitution, nonsynonymous status, sample frequency, and functional or biological impact where applicable.

**Supporting Information 4** Additional File 4. A summary of whole genome sequencing (WGS) results used in phylogenetic analysis. This table reports the relationship between samples within each cluster based on maximum‐likelihood trees, including details on clade and lineage concordance, and sequence‐level similarity or divergence.

**Supporting Information 5** Table S1. Detailed metadata and genomic features of the SARS‐CoV‐2 isolates analyzed in this study. The table integrates epidemiological, demographic, and sequencing‐related information with lineage classification and identified amino acid variations, providing additional context for the phylogenetic analyses and supporting the interpretation of transmission patterns presented in the main manuscript.

## Data Availability

The genomic consensus sequences generated in this study have been deposited in the GISAID database (https://www.gisaid.org/) and are publicly accessible to registered users. The sequences are available under the following accession numbers for the year 2020: EPI_ISL_19536597–19536598, EPI_ISL_19536809, EPI_ISL_19536847–19536848, EPI_ISL_19536850, EPI_ISL_19536875, EPI_ISL_19536895–19536896, EPI_ISL_19539150, EPI_ISL_19539227, EPI_ISL_19539249, EPI_ISL_19539483–19539484, EPI_ISL_19539499, EPI_ISL_19539531–19539537, EPI_ISL_19539555–19539556, EPI_ISL_19539560, and EPI_ISL_19539566–19539573. For the year 2021, the available accession numbers are EPI_ISL_19536897–19536899, EPI_ISL_19537083, EPI_ISL_19537185, EPI_ISL_19539081, EPI_ISL_19539250–19539252, EPI_ISL_19539284–19539285, EPI_ISL_19539301, EPI_ISL_19539331, EPI_ISL_19539354, EPI_ISL_19539467, EPI_ISL_19539563–19539565, and EPI_ISL_19539574–19539585. A publicly accessible dataset summary is also available via GISAID’s EpiSet DOI https://doi.org/10.55876/gis8.241118kt, which includes all 63 genome sequences and detailed metadata used in this study. This table is also included as Supporting Table S1. The data supporting the findings of this study are available within the article and supporting information. Due to ethical and confidentiality restrictions, raw sequencing reads are not publicly available but may be shared upon reasonable request to the corresponding author, subject to institutional approval.
