# Supplementary material for: Genomic and Phylogenetic Dissection of SARS‐CoV‐2 Transmission Networks in Healthcare Workers
Source: Int J Microbiol. 2026 Apr 21;2026:6610060. doi: 10.1155/ijm/6610060 (PMC13096929; doi:10.1155/ijm/6610060)
Supplement: Supplementary file 1 — Supporting Information 1 Additional File 1. A table presenting the detailed demographic characteristics and epidemiological cluster assignments of SARS‐CoV‐2–positive individuals included in the study. Cluster numbers reflect groupings based on filiation data, and information includes sample number, sex, age, and participant status. [file IJM-2026-6610060-s001.pdf]

Additional File 1

This table provides detailed demographic information and cluster assignments for the SARS-CoV-2 samples included in the study. The "Cluster no" column reflects groupings based on filiation data, indicating epidemiological links between cases. The table include the sample number, sex, age, and the status (e.g., healthcare worker) of each participant.

	Cluster no	Sample no	Sex	Status	Age
Included Samples	1	1	Male	Healthcare Worker	41
		2	Female	Healthcare Worker	26
		3	Female	Healthcare Worker	26
	2	4	Female	Healthcare Worker	29
		5	Male	Healthcare Worker	47
	3	6	Female	Healthcare Worker	22
		7	Female	Healthcare Worker	23
	4	8	Female	Healthcare Worker	36
		9	Female	Healthcare Worker	38
		64	Female	Healthcare Worker	25
	5	10	Female	Healthcare Worker	26
		11	Female	Healthcare Worker	28
		12	Female	Healthcare Worker	26
	6	13	Male	Healthcare Worker	24
		14	Male	Healthcare Worker	24
		15	Female	Healthcare Worker	24
	7	16	Male	Healthcare Worker	45
		17	Female	Family Member	20
		18	Female	Family Member	39
	8	19	Male	Healthcare Worker	44
		20	Female	Family Member	14
		21	Female	Family Member	8
		22	Female	Family Member	17
	9	23	Female	Healthcare Worker	38
		24	Female	Family Member	13
		25	Male	Family Member	42
		26	Female	Healthcare Worker	47
		27	Female	Healthcare Worker	22
	10	28	Male	Family Member	48
		29	Female	Family Member	42
		30	Male	Healthcare Worker	41
		31	Male	Healthcare Worker	37
		32	Male	Family Member	76
		33	Male	Family Member	58
		34	Male	Family Member	21
		35	Male	Healthcare Worker	41
		65	Male	Healthcare Worker	40
	11	36	Female	Healthcare Worker	25
		37	Male	Healthcare Worker	27
		38	Male	Healthcare Worker	26
	12	39	Female	Healthcare Worker	26
		40	Female	Healthcare Worker	27
		66	Female	Healthcare Worker	25
	13	41	Male	Healthcare Worker	27
		42	Male	Healthcare Worker	26
		43	Male	Healthcare Worker	26
	14	44	Male	Family Member	6
		45	Female	Family Member	26
		46	Male	Healthcare Worker	34
	15	47	Female	Family Member	23
		48	Male	Healthcare Worker	48
	16	49	Female	Healthcare Worker	42
		50	Male	Family Member	45
		51	Female	Family Member	15
	17	52	Female	Healthcare Worker	47
		53	Female	Healthcare Worker	49
		54	Female	Healthcare Worker	42
	18	55	Male	Family Member	81
		56	Female	Family Member	57
		57	Female	Healthcare Worker	48
	19	58	Female	Healthcare Worker	45
		59	Male	Family Member	49
		60	Male	Family Member	23
	20	61	Female	Healthcare Worker	45
		62	Male	Family Member	44
		63	Male	Family Member	10
Excluded Samples	21	67	Male	Healthcare Worker	55
		68	Male	Healthcare Worker	55
		69	Male	Healthcare Worker	28
		70	Female	Healthcare Worker	53
		71	Male	Healthcare Worker	26
		72	Male	Healthcare Worker	26
	22	73	Male	Healthcare Worker	21
		74	Male	Healthcare Worker	42
		75	Female	Healthcare Worker	34
	23	76	Female	Healthcare Worker	27
		77	Male	Healthcare Worker	26
		78	Female	Healthcare Worker	43
		79	Male	Family Member	55
	24	80	Female	Healthcare Worker	25
		81	Female	Healthcare Worker	23
		82	Female	Healthcare Worker	24
		83	Female	Healthcare Worker	23
	25	84	Male	Healthcare Worker	49
		85	Female	Healthcare Worker	35
	26	86	Female	Healthcare Worker	27
		87	Female	Healthcare Worker	35
	27	88	Female	Healthcare Worker	30
		89	Female	Healthcare Worker	33
	28	90	Male	Healthcare Worker	47
		91	Male	Family Member	6 ay
		92	Male	Family Member	16
		93	Female	Family Member	34
