# Supplementary material for: Genomic and Phylogenetic Dissection of SARS‐CoV‐2 Transmission Networks in Healthcare Workers
Source: Int J Microbiol. 2026 Apr 21;2026:6610060. doi: 10.1155/ijm/6610060 (PMC13096929; doi:10.1155/ijm/6610060)
Supplement: Supplementary file 2 — Supporting Information 2 Additional File 2. A comprehensive table of sequence analysis results from 63 individuals across 20 clusters. It includes demographic data, RT‐PCR Ct values, total reads, consensus length, unknown base percentages, average coverage, 10 × coverage, and reference coverage. It also contains clade and lineage classifications and metadata for sequences submitted to GISAID. [file IJM-2026-6610060-s002.pdf]

Additional File 2

This table provides detailed sequence analysis data from 63 individuals grouped into 20 clusters, incorporating demographic information such as cluster number, sample number, sex, age, and individual status (e.g., healthcare workers). Additionally, RT-PCR data, including Cycle Threshold (Ct) values, are presented. Sequence analysis results are also provided, showcasing key metrics such as total reads, consensus length, unknown bases, average coverage, 10x coverage, and reference coverage. Clade and lineage information for each sample is included, alongside the metadata for sequences uploaded to the GISAID database. The table highlights samples uploaded to the Miniseq platform with orange-colored rows, while uncolored rows represent samples uploaded to the Nextseq platform.

Cluster no	Sample no	Sex	Status	Age	Cycle threshold (cy5 channel)	Total Reads	Consensus Length (no N's)	Average Coverage	Coverage (10x) %	Reference Coverage %	N %	CLADE nextclade 2.5.0 (tree-2022-09-09)	Lineage
1	1	Male	Healthcare Worker	41	13	820.654	29903	1252	94,7	88	12	20A	B.1.469
	2	Female	Healthcare Worker	26	21	1.250.628	29903	1264	96,4	89	10,6	20A	B.1.469
	3	Female	Healthcare Worker	26	20	1.523.388	29903	2525	92,2	86	14,5	20A	B.1.469
2	4	Female	Healthcare Worker	29	23	866.546	29903	1177	91,9	84	16,2	20B	B.1.1
	5	Male	Healthcare Worker	47	23	1.328.638	29903	1297	96,6	88	11,6	20B	B.1.1
3	6	Female	Healthcare Worker ¥	22	22	2.101.492	29906	1333	96,9	92	8,3	20B	B.1.1
	7	Female	Healthcare Worker ¥	23	16	1.113.026	29906	1496	91,4	85	15,3	20B	B.1.1
4	8	Female	Healthcare Worker ¥	36	16	1.218.552	29903	1531	89,8	82	17,8	20B	B.1.1.181
	9	Female	Healthcare Worker ¥	38	17	1.525.896	29903	1716	98,7	95	5,3	20B	B.1.1
5	10	Female	Healthcare Worker ¥	26	16	3.149.940	29755	4397	99,1	98	1,5	20I (Alpha, V1)	B.1.1.7
	11	Female	Healthcare Worker ¥	28	18	4.179.316	29785	3757	96,8	95	5	20I (Alpha, V1)	B.1.1.7
	12	Female	Healthcare Worker ¥	26	22	5.291.092	29766	3476	97,4	95	4,8	20I (Alpha, V1)	B.1.1.7
6	13	Male	Healthcare Worker ¥	24	22	5.065.556	29763	4534	98,9	99	0.9	20I (Alpha, V1)	B.1.1.7
	14	Male	Healthcare Worker ¥	24	22	5.052.330	29763	3516	99,0	99	0,9	20I (Alpha, V1)	B.1.1.7
	15	Female	Healthcare Worker ¥	24	19	4.363.864	29763	4428	98,9	98	1,6	20I (Alpha, V1)	B.1.1.7
7	16	Male	Healthcare Worker ¥	45	17	4.786.490	29803	4757	98,1	96	3,6	20B	B.1.1
	17	Female	Family Member	20	17	2.943.182	29803	4987	98,7	98	1,7	20B	B.1.1
	18	Female	Family Member	39	15	2.613.528	29803	4336	99,0	98	1,4	20B	B.1.1
8	19	Male	Healthcare Worker	44	15	2.430.494	29627	3943	97,6	97	1,9	21J (Delta)	AY.126
	20	Female	Family Member	14	18	3.595.010	29772	4990	98,4	98	2	21J (Delta)	AY.126
	21	Female	Family Member	8	22	5.042.792	29772	5476	98,4	98	1,5	21J (Delta)	AY.126
	22	Female	Family Member	17	11	2.867.088	29627	5205	98,6	97	1,8	21J (Delta)	AY.126
9	23	Female	Healthcare Worker ¥	38	12	2.360.518	29627	3977	98,1	98	1,5	21J (Delta)	B.1.617.2
	24	Female	Family Member	13	14	3.028.716	29761	5113	99,2	98	1,1	21J (Delta)	B.1.617.2
	25	Male	Family Member	42	17	3.166.680	29761	4198	98,2	97	2,2	21J (Delta)	B.1.617.2
	26	Female	Healthcare Worker ¥	47	16	3.225.044	29761	5300	99,4	98	1,5	21J (Delta)	B.1.617.2
	27	Female	Healthcare Worker ¥	22	13	2.985.714	29772	5468	99,2	98	1,1	21J (Delta)	B.1.617.2
10	28#	Male	Family Member	48	14	2.658.792	29639	4623 (904)	97,47 (91,9)	96 (82)	3,6 (18,4)	20B	B.1.1
	29#	Female	Family Member	42	19	3.400.074	29639	5601 (1545)	99,34 (97,1)	98 (91)	1 (9,4)	20A	B.1
	30#	Male	Healthcare Worker	41	22	4.981.294	29639	5766 (1541)	99,34 (97,4)	99 (94)	0,3 (6,5)	20A	B.1
	31#	Male	Healthcare Worker	37	21	3.291.478	29773	5608 (1524)	98,39 (94,8)	97 (88)	2,6 (11,6)	20A	B.1
	32#	Male	Family Member	76	12	2.547.472	29639	4965 (1888)	98,01 (95,2)	96 (91)	3,2 (9,4)	20B	B.1.1
	33#	Male	Family Member	58	21	2.969.896	29639	5516 (1550)	99,15 (96,8)	98 (91)	1,1 (9,5)	20A	B.1
	34#	Male	Family Member	21	24	4.176.364	29784	5483 (1290)	99,34 (97,4)	99 (92)	0,7 (8,2)	20A	B.1
	35#	Male	Healthcare Worker	41	24	4.596.718	29784	4584 (1210)	99,45 (96,8)	99 (90)	0,7 (10)	20A	B.1
11	36#	Female	Healthcare Worker ¥	25	17	3.184.440	29792	4665,5 (1030)	97,37 (85,9)	93 (74)	6 (25,7)	20A	B.1.160
	37#	Male	Healthcare Worker ¥	27	18	3.388.956	29639	4684,3 (882)	98,57 (94)	97 (85)	1,9 (15,2)	20A	B.1.160
	38#	Male	Healthcare Worker ¥	26	16	3.927.786	29792	5152,2 (1428)	98,75 (94,3)	97 (86)	2,4 (13,8)	20A	B.1.362
12	39#	Female	Healthcare Worker ¥	26	17	3.433.976	29639	5313,3 (1674)	98,47 (97,3)	97 (93)	1,2 (6,8)	20A	B.1.36
	40#	Female	Healthcare Worker ¥	27	14	2.901.262	29639	5259,6 (1213)	98,7 (97)	97 (93)	1,2 (7,3)	20A	B.1.36
13	41	Male	Healthcare Worker ¥	27	12	3.419.644	29772	5078	99,1	98	1	21J (Delta)	AY.46
	42	Male	Healthcare Worker ¥	26	20	4.724.222	29772	3806	98,6	98	1,1	21J (Delta)	AY.46
	43	Male	Healthcare Worker ¥	26	17	2.709.048	29772	4969	99,0	98	1	21J (Delta)	AY.78
14	44	Male	Family Member	6	15	3.146.318	29795	5238	99,4	99	0,6	20A	B.1
	45	Female	Family Member	26	19	3.967.828	29803	4779	96,5	93	6,3	20A	B.1
	46	Male	Healthcare Worker ¥	34	18	3.412.210	29784	5249	99,4	99	0,3	20A	B.1
15	47	Female	Family Member	23	11	2.465.844	29803	4715	98,9	98	0,8	20A	B.1.160
	48	Male	Healthcare Worker	48	18	3.494.262	29803	5119	98,8	98	1,9	20A	B.1.160
16	49	Female	Healthcare Worker ¥	42	17	4.002.096	29803	5208	98,4	98	2,2	20B	B.1.1.28
	50	Male	Family Member	45	19	3.461.920	29784	3455	99,8	99	0,5	20B	B.1.1.28
	51	Female	Family Member	15	16	2.565.260	29784	2788	96,6	93	6,7	20B	B.1.1.28
17	52	Female	Healthcare Worker	47	18	4.214.778	29772	5464	98,9	98	1,2	21J (Delta)	AY.121
	53	Female	Healthcare Worker	49	17	3.175.508	29772	5520	98,7	98	1,5	21J (Delta)	AY.121
	54	Female	Healthcare Worker	42	19	3.561.906	29772	4689	98,3	98	1,5	21J (Delta)	AY.121
18	55	Male	Family Member	81	11	2.587.798	29766	4996	98,7	97	1,7	21J (Delta)	AY.126
	56	Female	Family Member	57	12	2.437.418	29766	4709	98,2	97	1,6	21J (Delta)	AY.126
	57	Female	Healthcare Worker	48	14	3.233.574	29766	5218	98,4	97	1,7	21J (Delta)	AY.126
19	58	Female	Healthcare Worker ¥	45	21	4.031.386	29772	4834	98,0	97	2,2	21J (Delta)	AY.126
	59	Male	Family Member	49	12	4.196.716	29772	5086	98,7	97	1,9	21J (Delta)	AY.126
	60	Male	Family Member	23	17	2.569.736	29772	4494	98,0	97	2	21J (Delta)	AY.126
20	61	Female	Healthcare Worker ¥	45	19	5.548.376	29772	5260	98,9	98	1	21J (Delta)	AY.121
	62	Male	Family Member	44	17	3.280.812	29772	4975	98,8	98	1	21J (Delta)	AY.121
	63	Male	Family Member	10	12	2.861.662	29772	4832	99,1	98	1	21J (Delta)	AY.121

Sample no	GISAID Sample Metadata								
	Virus name	Accession ID	Collection date	Location	Host	Sampling strategy	Patient status	Passage	Specimen
1	hCoV-19/Turkey/DEU-S1/2020	EPI_ISL_19536597	2020-09-04	Europe / Turkey	Human	Outbreak investigation	unknown	Original	Nasopharyngeal swab
2	hCoV-19/Turkey/DEU-S2/2020	EPI_ISL_19536598	2020-09-09	Europe / Turkey	Human	Outbreak investigation	unknown	Original	Nasopharyngeal swab
3	hCoV-19/Turkey/DEU-S3/2020	EPI_ISL_19536809	2020-09-10	Europe / Turkey	Human	Outbreak investigation	unknown	Original	Nasopharyngeal swab
4	hCoV-19/Turkey/DEU-S4/2020	EPI_ISL_19536847	2020-10-09	Europe / Turkey	Human	Outbreak investigation	unknown	Original	Nasopharyngeal swab
5	hCoV-19/Turkey/DEU-S5/2020	EPI_ISL_19536848	2020-10-16	Europe / Turkey	Human	Outbreak investigation	unknown	Original	Nasopharyngeal swab
6	hCoV-19/Turkey/DEU-S6/2020	EPI_ISL_19536850	2020-11-07	Europe / Turkey	Human	Outbreak investigation	unknown	Original	Nasopharyngeal swab
7	hCoV-19/Turkey/DEU-S7/2020	EPI_ISL_19536875	2020-11-16	Europe / Turkey	Human	Outbreak investigation	unknown	Original	Nasopharyngeal swab
8	hCoV-19/Turkey/DEU-S8/2020	EPI_ISL_19536895	2020-11-11	Europe / Turkey	Human	Outbreak investigation	unknown	Original	Nasopharyngeal swab
9	hCoV-19/Turkey/DEU-S9/2020	EPI_ISL_19536896	2020-11-12	Europe / Turkey	Human	Outbreak investigation	unknown	Original	Nasopharyngeal swab
10	hCoV-19/Turkey/DEU-S10/2021	EPI_ISL_19536897	2021-04-20	Europe / Turkey	Human	Outbreak investigation	unknown	Original	Nasopharyngeal swab
11	hCoV-19/Turkey/DEU-S11/2021	EPI_ISL_19536898	2021-04-20	Europe / Turkey	Human	Outbreak investigation	unknown	Original	Nasopharyngeal swab
12	hCoV-19/Turkey/DEU-S12/2021	EPI_ISL_19536899	2021-04-22	Europe / Turkey	Human	Outbreak investigation	unknown	Original	Nasopharyngeal swab
13	hCoV-19/Turkey/DEU-S13/2021	EPI_ISL_19537083	2021-05-17	Europe / Turkey	Human	Outbreak investigation	unknown	Original	Nasopharyngeal swab
14	hCoV-19/Turkey/DEU-S14/2021	EPI_ISL_19537185	2021-05-20	Europe / Turkey	Human	Outbreak investigation	unknown	Original	Nasopharyngeal swab
15	hCoV-19/Turkey/DEU-S15/2021	EPI_ISL_19539081	2021-05-25	Europe / Turkey	Human	Outbreak investigation	unknown	Original	Nasopharyngeal swab
16	hCoV-19/Turkey/DEU-S16/2020	EPI_ISL_19539150	2020-11-03	Europe / Turkey	Human	Outbreak investigation	unknown	Original	Nasopharyngeal swab
17	hCoV-19/Turkey/DEU-S17/2020	EPI_ISL_19539227	2020-11-11	Europe / Turkey	Human	Outbreak investigation	unknown	Original	Nasopharyngeal swab
18	hCoV-19/Turkey/DEU-S18/2020	EPI_ISL_19539249	2020-11-11	Europe / Turkey	Human	Outbreak investigation	unknown	Original	Nasopharyngeal swab
19	hCoV-19/Turkey/DEU-S19/2021	EPI_ISL_19539250	2021-09-22	Europe / Turkey	Human	Outbreak investigation	unknown	Original	Nasopharyngeal swab
20	hCoV-19/Turkey/DEU-S20/2021	EPI_ISL_19539251	2021-09-20	Europe / Turkey	Human	Outbreak investigation	unknown	Original	Nasopharyngeal swab
21	hCoV-19/Turkey/DEU-S21/2021	EPI_ISL_19539252	2021-09-23	Europe / Turkey	Human	Outbreak investigation	unknown	Original	Nasopharyngeal swab
22	hCoV-19/Turkey/DEU-S22/2021	EPI_ISL_19539284	2021-09-24	Europe / Turkey	Human	Outbreak investigation	unknown	Original	Nasopharyngeal swab
23	hCoV-19/Turkey/DEU-S23/2021	EPI_ISL_19539285	2021-09-25	Europe / Turkey	Human	Outbreak investigation	unknown	Original	Nasopharyngeal swab
24	hCoV-19/Turkey/DEU-S24/2021	EPI_ISL_19539301	2021-09-28	Europe / Turkey	Human	Outbreak investigation	unknown	Original	Nasopharyngeal swab
25	hCoV-19/Turkey/DEU-S25/2021	EPI_ISL_19539331	2021-10-04	Europe / Turkey	Human	Outbreak investigation	unknown	Original	Nasopharyngeal swab
26	hCoV-19/Turkey/DEU-S26/2021	EPI_ISL_19539354	2021-09-25	Europe / Turkey	Human	Outbreak investigation	unknown	Original	Nasopharyngeal swab
27	hCoV-19/Turkey/DEU-S27/2021	EPI_ISL_19539467	2021-09-26	Europe / Turkey	Human	Outbreak investigation	unknown	Original	Nasopharyngeal swab
28#	hCoV-19/Turkey/DEU-S28/2020	EPI_ISL_19539483	2020-04-11	Europe / Turkey	Human	Research - specific population	unknown	Original	Nasopharyngeal swab
29#	hCoV-19/Turkey/DEU-S29/2020	EPI_ISL_19539484	2020-04-14	Europe / Turkey	Human	Research - specific population	unknown	Original	Nasopharyngeal swab
30#	hCoV-19/Turkey/DEU-S30/2020	EPI_ISL_19539499	2020-04-14	Europe / Turkey	Human	Research - specific population	unknown	Original	Nasopharyngeal swab
31#	hCoV-19/Turkey/DEU-S31/2020	EPI_ISL_19539531	2020-04-15	Europe / Turkey	Human	Research - specific population	unknown	Original	Nasopharyngeal swab
32#	hCoV-19/Turkey/DEU-S32/2020	EPI_ISL_19539532	2020-04-19	Europe / Turkey	Human	Research - specific population	unknown	Original	Nasopharyngeal swab
33#	hCoV-19/Turkey/DEU-S33/2020	EPI_ISL_19539533	2020-04-14	Europe / Turkey	Human	Research - specific population	unknown	Original	Nasopharyngeal swab
34#	hCoV-19/Turkey/DEU-S34/2020	EPI_ISL_19539534	2020-04-15	Europe / Turkey	Human	Research - specific population	unknown	Original	Nasopharyngeal swab
35#	hCoV-19/Turkey/DEU-S35/2020	EPI_ISL_19539535	2020-04-16	Europe / Turkey	Human	Research - specific population	unknown	Original	Nasopharyngeal swab
36#	hCoV-19/Turkey/DEU-S36/2020	EPI_ISL_19539536	2020-11-20	Europe / Turkey	Human	Research - specific population	unknown	Original	Nasopharyngeal swab
37#	hCoV-19/Turkey/DEU-S37/2020	EPI_ISL_19539537	2020-11-20	Europe / Turkey	Human	Research - specific population	unknown	Original	Nasopharyngeal swab
38#	hCoV-19/Turkey/DEU-S38/2020	EPI_ISL_19539555	2020-11-25	Europe / Turkey	Human	Research - specific population	unknown	Original	Nasopharyngeal swab
39#	hCoV-19/Turkey/DEU-S39/2020	EPI_ISL_19539556	2020-12-03	Europe / Turkey	Human	Research - specific population	unknown	Original	Nasopharyngeal swab
40#	hCoV-19/Turkey/DEU-S40/2020	EPI_ISL_19539560	2020-12-03	Europe / Turkey	Human	Research - specific population	unknown	Original	Nasopharyngeal swab
41	hCoV-19/Turkey/DEU-S41/2021	EPI_ISL_19539563	2021-08-06	Europe / Turkey	Human	Research - specific population	unknown	Original	Nasopharyngeal swab
42	hCoV-19/Turkey/DEU-S42/2021	EPI_ISL_19539564	2021-08-07	Europe / Turkey	Human	Research - specific population	unknown	Original	Nasopharyngeal swab
43	hCoV-19/Turkey/DEU-S43/2021	EPI_ISL_19539565	2021-08-11	Europe / Turkey	Human	Research - specific population	unknown	Original	Nasopharyngeal swab
44	hCoV-19/Turkey/DEU-S44/2020	EPI_ISL_19539566	2020-08-20	Europe / Turkey	Human	Research - specific population	unknown	Original	Nasopharyngeal swab
45	hCoV-19/Turkey/DEU-S45/2020	EPI_ISL_19539567	2020-08-21	Europe / Turkey	Human	Research - specific population	unknown	Original	Nasopharyngeal swab
46	hCoV-19/Turkey/DEU-S46/2020	EPI_ISL_19539568	2020-08-24	Europe / Turkey	Human	Research - specific population	unknown	Original	Nasopharyngeal swab
47	hCoV-19/Turkey/DEU-S47/2020	EPI_ISL_19539569	2020-10-29	Europe / Turkey	Human	Research - specific population	unknown	Original	Nasopharyngeal swab
48	hCoV-19/Turkey/DEU-S48/2020	EPI_ISL_19539570	2020-11-01	Europe / Turkey	Human	Research - specific population	unknown	Original	Nasopharyngeal swab
49	hCoV-19/Turkey/DEU-S49/2020	EPI_ISL_19539571	2020-10-30	Europe / Turkey	Human	Research - specific population	unknown	Original	Nasopharyngeal swab
50	hCoV-19/Turkey/DEU-S50/2020	EPI_ISL_19539572	2020-10-31	Europe / Turkey	Human	Research - specific population	unknown	Original	Nasopharyngeal swab
51	hCoV-19/Turkey/DEU-S51/2020	EPI_ISL_19539573	2020-11-03	Europe / Turkey	Human	Research - specific population	unknown	Original	Nasopharyngeal swab
52	hCoV-19/Turkey/DEU-S52/2021	EPI_ISL_19539574	2021-09-10	Europe / Turkey	Human	Research - specific population	unknown	Original	Nasopharyngeal swab
53	hCoV-19/Turkey/DEU-S53/2021	EPI_ISL_19539575	2021-09-17	Europe / Turkey	Human	Research - specific population	unknown	Original	Nasopharyngeal swab
54	hCoV-19/Turkey/DEU-S54/2021	EPI_ISL_19539576	2021-09-20	Europe / Turkey	Human	Research - specific population	unknown	Original	Nasopharyngeal swab
55	hCoV-19/Turkey/DEU-S55/2021	EPI_ISL_19539577	2021-10-22	Europe / Turkey	Human	Research - specific population	unknown	Original	Nasopharyngeal swab
56	hCoV-19/Turkey/DEU-S56/2021	EPI_ISL_19539578	2021-10-30	Europe / Turkey	Human	Research - specific population	unknown	Original	Nasopharyngeal swab
57	hCoV-19/Turkey/DEU-S57/2021	EPI_ISL_19539579	2021-11-01	Europe / Turkey	Human	Research - specific population	unknown	Original	Nasopharyngeal swab
58	hCoV-19/Turkey/DEU-S58/2021	EPI_ISL_19539580	2021-11-01	Europe / Turkey	Human	Research - specific population	unknown	Original	Nasopharyngeal swab
59	hCoV-19/Turkey/DEU-S59/2021	EPI_ISL_19539581	2021-11-01	Europe / Turkey	Human	Research - specific population	unknown	Original	Nasopharyngeal swab
60	hCoV-19/Turkey/DEU-S60/2021	EPI_ISL_19539582	2021-11-05	Europe / Turkey	Human	Research - specific population	unknown	Original	Nasopharyngeal swab
61	hCoV-19/Turkey/DEU-S61/2021	EPI_ISL_19539583	2021-11-10	Europe / Turkey	Human	Research - specific population	unknown	Original	Nasopharyngeal swab
62	hCoV-19/Turkey/DEU-S62/2021	EPI_ISL_19539584	2021-11-10	Europe / Turkey	Human	Research - specific population	unknown	Original	Nasopharyngeal swab
63	hCoV-19/Turkey/DEU-S63/2021	EPI_ISL_19539585	2021-11-11	Europe / Turkey	Human	Research - specific population	unknown	Original	Nasopharyngeal swab

#: Samples sequenced in both Miniseq and Nextseq.   ¥: Clinical unit.   N: Unknow base
