# Supplementary material for: Genomic and Phylogenetic Dissection of SARS‐CoV‐2 Transmission Networks in Healthcare Workers
Source: Int J Microbiol. 2026 Apr 21;2026:6610060. doi: 10.1155/ijm/6610060 (PMC13096929; doi:10.1155/ijm/6610060)
Supplement: Supplementary file 3 — Supporting Information 3 Additional File 3. A table summarizing all nucleotide variations identified in the sequenced SARS‐CoV‐2 genomes. Each entry includes the gene, region, type of variation, amino acid substitution, nonsynonymous status, sample frequency, and functional or biological impact where applicable. [file IJM-2026-6610060-s003.pdf]

Additional File 3

This table details the nucleotide variations observed in the SARS-CoV-2 samples. It includes columns specifying the gene, region, type of variation, amino acid (AA) substitution, whether the variation is non-synonymous, and the sample count for each variation. Additionally, the table highlights the function or impact of some key mutations, offering insights into their potential significance. This summary provides an overview of the genetic variations found in the analyzed samples.

Gene	Region	Type	Reference	Allelle	AA Substitution	Non-synonymous	Sample count	Function / Impact [38,39]
5'UTR	241	SNV	C	T		-	63	Transmission
	210	SNV	G	T		-	24	
	174	SNV	G	T		-	4	
	219	SNV	G	C		-	4	
	61	SNV	G	A		-	1	
ORF1ab	3037	SNV	C	T		-	63	Transmission
	14408	SNV	C	T	P4715L	+	63	Transmission
	6402	SNV	C	T	P2046L	+	25	
	8986	SNV	C	T		-	24	
	11332	SNV	A	G		-	24	
	4181	SNV	G	T	A1306S	+	24	
	7124	SNV	C	T	P2287S	+	24	
	9053	SNV	G	T	V2930L	+	24	
	10029	SNV	C	T	T3255I	+	24	
	15451	SNV	G	A	G5063S	+	24	
	16466	SNV	C	T	P5401L	+	24	
	19220	SNV	C	T	A6319V	+	24	
	11201	SNV	A	G	T3646A	+	23	
	21137	SNV	A	G	K6958R	+	16	
	313	SNV	C	T		-	11	
	14925	SNV	C	T		-	10	
	18877	SNV	C	T		-	9	
	913	SNV	C	T		-	6	
	5986	SNV	C	T		-	6	
	11288..11296	Deletion	TCTGGTTTT	-	S3675-,G3676-,F3677-	+	6	
	13168	SNV	C	T		-	6	
	14676	SNV	C	T		-	6	
	15279	SNV	C	T		-	6	
	16176	SNV	T	C		-	6	
	5388	SNV	C	A	A1708D	+	6	
	6954	SNV	T	C	I2230T	+	6	
	10138	SNV	C	T		-	5	
	11653	SNV	C	T		-	5	
	12139	SNV	T	C		-	5	
	3267	SNV	C	T	T1001I	+	5	
	12513	SNV	C	T	T4083M	+	5	
	19549	SNV	G	T	A6429S	+	5	
	683	SNV	C	T		-	4	
	5629	SNV	G	T		-	4	
	6196	SNV	C	T		-	4	
	11497	SNV	C	T		-	4	
	384	SNV	C	T	S40L	+	4	
	3951	SNV	A	G	D1229G	+	4	
ORF1ab	6701	SNV	C	T	L2146F	+	4	
	9526	SNV	G	T	M3087I	+	4	
	9652	SNV	G	T	M3129I	+	4	
	13993	SNV	G	T	A4577S	+	4	
	15243	SNV	G	T	M4993I	+	4	
	15766	SNV	G	T	V5168L	+	4	
	17019	SNV	G	T	E5585D	+	4	
	19732	SNV	G	T	V6490F	+	4	
	4540	SNV	C	T		-	3	
	4999	SNV	C	T		-	3	
	5002	SNV	C	T		-	3	
	6037	SNV	C	T		-	3	
	7675	SNV	G	T		-	3	
	7732	SNV	C	T		-	3	
	10657	SNV	A	G		-	3	
	11455	SNV	C	T		-	3	
	14445	SNV	T	A		-	3	
	15096	SNV	T	C		-	3	
	15324	SNV	C	T		-	3	
	16260	SNV	C	T		-	3	
	3045	SNV	C	T	P927L	+	3	
	3239	SNV	G	A	D992N	+	3	
	5093	SNV	C	T	H1610Y	+	3	
	6354	SNV	C	T	S2030L	+	3	
	6807	SNV	C	T	T2181I	+	3	
	7306	SNV	G	T	M2347I	+	3	
	7712	SNV	C	T	P2483S	+	3	
	17142	SNV	T	A		-	3	
	8149	SNV	T	A	N2628K	+	3	
	9344	SNV	C	T	L3027F	+	3	
	10448	SNV	C	T	P3395S	+	3	
	17688	SNV	T	C		-	3	
	17721	SNV	G	T		-	3	
	10818	SNV	C	T	A3518V	+	3	
	18084	SNV	C	T		-	3	
	10835	SNV	G	A	V3524I	+	3	
	11083	SNV	G	T	L3606F	+	3	
	14854	SNV	G	A	V4864I	+	3	

Gene	Region	Type	Reference	Allele	AA Substitution	Non-synonymous	Sample count	Function / Impact [38,39]
ORF1ab	15436	SNV	A	G	M5058V	+	3	
	20946	SNV	C	T		-	3	
	15656	SNV	C	T	T5131I	+	3	
	16468	SNV	C	T	P5402S	+	3	
	16558	SNV	G	T	A5432S	+	3	
	16795	SNV	G	T	V5511L	+	3	
	17615	SNV	A	G	K5784R	+	3	
	17750	SNV	C	T	A5829V	+	3	
	21334	SNV	G	T	A7024S	+	3	
	21512	SNV	A	G	N7083S	+	3	
	823	SNV	C	T		-	2	
	4543	SNV	C	T		-	2	
	6982	SNV	C	T		-	2	
	7148	SNV	T	C		-	2	
	8017	SNV	G	T		-	2	
	593	SNV	C	T	H110Y	+	2	
	625	SNV	G	T	K120N	+	2	
	1862	SNV	C	T	L533F	+	2	
	2445	SNV	C	T	T727I	+	2	
	3768	SNV	C	T	T1168I	+	2	
	9826	SNV	A	G		-	2	
	5264	SNV	A	G	I1667V	+	2	
	10156	SNV	C	T		-	2	
	6393	SNV	A	G	D2043G	+	2	
	6413	SNV	G	A	E2050K	+	2	
	6638	SNV	C	T	H2125Y	+	2	
	7042	SNV	G	T	M2259I	+	2	
	9188	SNV	G	T	V2975L	+	2	
	9584	SNV	G	A	V3107I	+	2	
	10335	SNV	C	T	A3357V	+	2	
	10977	SNV	C	T	A3571V	+	2	
	11605	SNV	G	A	M3780I	+	2	
	12075	SNV	A	G	N3937S	+	2	
	11704	SNV	C	T		-	2	
	11812	SNV	C	A		-	2	
	12202	SNV	G	T	K3979N	+	2	
	14318	SNV	C	T	T4685I	+	2	
	15569	SNV	C	T	T5102I	+	2	
	15654	SNV	C	T		-	2	
	19186	SNV	C	T		-	2	
	19524	SNV	C	T		-	2	
	20469	SNV	G	A		-	2	
	1059	SNV	C	T	T265I	+	1	
	1191	SNV	C	T	P309L	+	1	
	2117	SNV	G	A	G618S	+	1	
	3261	SNV	C	T	T999I	+	1	
	4285	SNV	G	T	E1340D	+	1	
	5691	SNV	C	T	A1809V	+	1	
	10323	SNV	A	G	K3353R	+	1	
	10328	SNV	G	A	D3355N	+	1	
	13188	SNV	T	C	I4308T	+	1	
	3058	SNV	A	G		-	1	
	13388	SNV	A	G	M4375V	+	1	
	14648	SNV	A	G	N4795S	+	1	
	14768	SNV	C	T	A4835V	+	1	
	3268	SNV	T	C		-	1	
	14874	SNV	G	T	K4870N	+	1	
	16726	SNV	C	T	H5488Y	+	1	
	17035	SNV	G	T	A5591S	+	1	
	17211	SNV	G	T	L5649F	+	1	
	17678	SNV	C	T	T5805M	+	1	
	19859	SNV	C	T	A6532V	+	1	
	20132	SNV	C	T	A6623V	+	1	
	20402	SNV	C	T	S6723L	+	1	
	21034	SNV	C	T	L6924F	+	1	
	5512	SNV	C	T		-	1	
	21076	SNV	A	G	T6938A	+	1	
	21110	SNV	C	T	T6949I	+	1	
	7393	SNV	G	T		-	1	
	10834	SNV	C	T		-	1	
	11173	SNV	C	T		-	1	
	15372	SNV	G	T		-	1	
	15738	SNV	C	T		-	1	
	16221	SNV	G	T		-	1	
	16353	SNV	T	C		-	1	
	16362	SNV	C	T		-	1	
	17427	SNV	G	T		-	1	
	18690	SNV	T	C		-	1	
	18744	SNV	C	T		-	1	
	19839	SNV	T	C		-	1	
	20037	SNV	T	C		-	1	
S	23403	SNV	A	G	D614G	+	63	S1/S2 junction, transmission, infectivity
	22029..22034	Deletion	AGTTCA	-	E158G	+	24	Immune evasion, infectivity
	21618	SNV	C	G	T19R	+	24	
	21987	SNV	G	A	G142D	+	24	Immune evasion
	22917	SNV	T	G	L452R	+	24	Receptor binding, immune evasion
	23604	SNV	C	G	P681R	+	24	Furin cleavage , transmission
	24410	SNV	G	A	D950N	+	24	Membrane fusion
	22995	SNV	C	A	T478K	+	22	Receptor binding
	24110	SNV	A	C	I850L	+	10	
	21765..21770	Deletion	TACATG	-	H69-, V70-	+	6	
	21991..21993	Deletion	TTA	-	Y144-	+	6	
	23557	SNV	C	T		-	6	
	23063	SNV	A	T	N501Y	+	6	Receptor binding
	23271	SNV	C	A	A570D	+	6	
	23709	SNV	C	T	T716I	+	6	
	24506	SNV	T	G	S982A	+	6	
	24914	SNV	G	C	D1118H	+	6	
	24445	SNV	G	T		-	5	
	21712	SNV	A	T		-	4	
	24274	SNV	T	C		-	4	
	23604	SNV	C	A	P681H	+	4	
	22088	SNV	C	T	L176F	+	3	
	22094	SNV	G	A	D178N	+	3	

Gene	Region	Type	Reference	Allelle	AA Substitution	Non-synonymous	Sample count	Function / Impact
S	22403	SNV	G	C	E281Q	+	3	
	23031	SNV	T	C	F490S	+	3	
	23525	SNV	C	T	H655Y	+	3	
	24099	SNV	C	T	A846V	+	3	
	24124	SNV	G	T	K854N	+	3	
	24348	SNV	G	T	S929I	+	3	
	25088	SNV	G	T	V1176F	+	3	
	23950	SNV	T	C		-	3	
	24109	SNV	C	T		-	3	
	22992	SNV	G	A	S477N	+	2	
	24402	SNV	A	T	K947I	+	2	
	24712	SNV	G	T	M1050I	+	2	
	24751	SNV	G	T	L1063F	+	2	
	24872	SNV	G	T	V1104L	+	2	
	22444	SNV	C	T		-	2	
	22543	SNV	T	A		-	2	
	22801	SNV	G	T		-	2	
	23059	SNV	C	T		-	2	
	23191	SNV	C	T		-	2	
	24616	SNV	C	T		-	2	
	21575	SNV	C	T	L5F	+	1	
	21627	SNV	C	T	T22I	+	1	
	22299	SNV	G	A	R246K	+	1	
	23042	SNV	T	C	S494P	+	1	
	24000	SNV	G	T	S813I	+	1	
	22624	SNV	C	T		-	1	
	22981	SNV	T	C		-	1	
	24124	SNV	G	A		-	1	
	25156	SNV	C	T		-	1	
ORF3a	25469	SNV	C	T	S26L	+	24	
	25439	SNV	A	C	K16T	+	10	
	25563	SNV	G	T	Q57H	+	10	
	25996	SNV	G	T	V202L	+	6	
	25775	SNV	G	T	W128L	+	4	
	25710	SNV	C	T		-	4	
	25844	SNV	C	T	T151I	+	3	
	25855	SNV	G	T	D155Y	+	3	
	26069	SNV	A	C	E226A	+	3	
	25413	SNV	C	T		-	3	
	25440	SNV	G	C	K16N	+	2	
	25555	SNV	G	T	V55F	+	2	
	25613	SNV	C	T	S74F	+	2	
	26191	SNV	C	T	P267S	+	2	
	25902	SNV	T	G		-	2	
	25528	SNV	C	T	L46F	+	2	
	25546	SNV	C	T	L52F	+	1	
	25970..25971	MNV	GG	TA	W193L	+	1	
ORF7a	27752	SNV	C	T	T120I	+	22	
	27638	SNV	T	C	V82A	+	10	
	27463	SNV	G	T	V24F	+	3	
	27692..27697	Deletion	TTTTTC	-	F101-, L102-	+	3	
	27707	SNV	C	T	A105V	+	2	
	27494	SNV	C	T	P34L	+	1	
	27613	SNV	G	T	V74F	+	1	
ORF7b	27874	SNV	C	T	T40I	+	24	
	27876..27877	MNV	TG	GT	C41V	+	3	
M	26767	SNV	T	C	I82T	+	24	
	26735	SNV	C	T		-	9	
	26876	SNV	T	C		-	4	
	27131	SNV	C	T		-	3	
	26527	SNV	C	T	A2V	+	2	
	26985	SNV	C	T	H155Y	+	1	
ORF6	27346	SNV	T	C	Y49H	+	2	
N	29402	SNV	G	T	D377Y	+	26	
	28461	SNV	A	G	D63G	+	24	
	28881	SNV	G	T	R203M	+	24	
	28916	SNV	G	T	G215C	+	24	
	28881..28883	MNV	GGG	AAC	R203_G204delinsKR (R203K, G204R)	+	20	
	28280..28282	MNV	GAT	CTA	D3L	+	6	
	28977	SNV	C	T	S235F	+	6	
	28975	SNV	G	C	M234I	+	4	
	29399	SNV	G	A	A376T	+	4	
	28346	SNV	G	T	G25C	+	3	
	28655	SNV	G	A	D128N	+	3	
	28854	SNV	C	T	S194L	+	3	
	28866	SNV	C	T	T198I	+	3	
	28881	SNV	G	A	R203K	+	3	
	29527	SNV	G	T	Q418H	+	3	
	28396	SNV	G	A		-	3	
	28396	SNV	G	T		-	3	
	28594	SNV	A	G		-	3	
	29164	SNV	T	C		-	3	
	28806	SNV	G	T	G178V	+	2	
	29468	SNV	G	C	D399H	+	2	
	28321	SNV	G	T		-	2	
	29119	SNV	C	T		-	2	
	29149	SNV	C	T		-	2	
	28311	SNV	C	T	P13L	+	1	
	28690	SNV	G	T	L139F	+	1	
	29200	SNV	C	T		-	1	
	29227	SNV	G	A		-	1	
N-ORF8	28271	Deletion	A	-		-	30	
ORF8	28248..28253	Deletion	GATTTC	-	D119-, F120-	+	23	
	27972	SNV	C	T	Q27*	+	6	
	28048	SNV	G	T	R52I	+	6	
	28095	SNV	A	T	K68*	+	2	
ORF10	29596	SNV	A	G	I13M	+	3	
3'UTR	29742	SNV	G	T		-	17	
	29744	SNV	G	T		-	1	

AA: Amino Acid, Del: Deletion, MNV: Multi-Nucleotide Variant, ORF: Open Reading Frame, SNV: Single Nucleotide Variant, UTR: Un-translated Region
