# Supplementary material for: Genomic and Phylogenetic Dissection of SARS‐CoV‐2 Transmission Networks in Healthcare Workers
Source: Int J Microbiol. 2026 Apr 21;2026:6610060. doi: 10.1155/ijm/6610060 (PMC13096929; doi:10.1155/ijm/6610060)
Supplement: Supplementary file 4 — Supporting Information 4 Additional File 4. A summary of whole genome sequencing (WGS) results used in phylogenetic analysis. This table reports the relationship between samples within each cluster based on maximum‐likelihood trees, including details on clade and lineage concordance, and sequence‐level similarity or divergence. [file IJM-2026-6610060-s004.pdf]

Additional File 4

This table presents the results of whole genome sequencing (WGS) analyses for samples from 20 clusters. The table includes columns for cluster number, sample number, status, whole genome sequencing results, clade and lineage information, and the relationship level in the maximum likelihood (ML) phylogenetic tree. All clusters were evaluated, and the samples highlighted in red indicate sequences that were not consistent with filiation analysis.

Cluster no	Sample no	Status	Whole Genome Sequencing	Clade & Lineage	ML Tree Location Relationship Level
1	1	Healthcare Worker	Identical variation	Same clade & lineage	> %95 very strong
	2	Healthcare Worker	1 SNV difference [§]		
	3	Healthcare Worker	1 SNV difference [§]		
2	4	Healthcare Worker	1 SNV difference [§]	Same clade & lineage	> %95 very strong
	5	Healthcare Worker			
3	6	Healthcare Worker	1 SNV difference[§]	Same clade & lineage	> %95 very strong
	7	Healthcare Worker			
4	8	Healthcare Worker	>2 SNV difference	Same clade, different lineage	Different location
	9	Healthcare Worker			
5	10	Healthcare Worker	Identical variation	Same clade & lineage	> %95 very strong
	11	Healthcare Worker	3 SNV difference[§]		
	12	Healthcare Worker	3 SNV difference[§]		
6	13	Healthcare Worker	Identical variation	Same clade & lineage	> %95 very strong
	14	Healthcare Worker	Identical variation		
	15	Healthcare Worker	2 SNV difference		
7	16	Healthcare Worker	1 SNV difference[§]	Same clade & lineage	> %95 very strong
	17	Family Member	Identical variation		
	18	Family Member	Identical variation		
8	19	Healthcare Worker	Identical variation	Same clade & lineage	> %95 very strong
	20	Family Member	Identical variation		
	21	Family Member	Identical variation		
	22	Family Member	1 SNV difference		
9	23	Healthcare Worker	1 SNV difference[§]	Same clade & lineage	> %95 very strong
	24	Family Member	1 SNV difference[§]		
	25	Family Member	1 SNV difference[§]		
	26	Healthcare Worker	Identical variation		
	27	Healthcare Worker	1 SNV difference		
10	28	Family Member	1 MNV difference	Different Clade& lineage	Different location
	29	Family Member	Identical variation	Same clade & lineage	< %75 weak
	30	Healthcare Worker	Identical variation		< %75 weak
	31	Healthcare Worker	1 SNV difference		< %75 weak
	32	Family Member	1 MNV difference	Different Clade& lineage	Different location
	33	Family Member	1 SNV difference	Same clade & lineage	< %75 weak
	34	Family Member	Identical variation		< %75 weak
	35	Healthcare Worker	Identical variation		< %75 weak
11	36	Healthcare Worker	1 SNV difference[§]	Same clade & lineage	> %95 very strong
	37	Healthcare Worker			
	38	Healthcare Worker	>2 SNV difference	Same clade, different lineage	Different location
12	39	Healthcare Worker	Identical variation	Same clade & lineage	>%95 very strong
	40	Healthcare Worker			
13	41	Healthcare Worker	Identical variation	Dal ve Soy ortak	%75-95 strong
	42	Healthcare Worker	1 SNV difference		
	43	Healthcare Worker	>2 SNV difference	Same clade, different lineage	Different location
14	44	Family Member	Identical variation	Same clade & lineage	> %95 very strong
	45	Family Member	1 SNV difference[§]		
	46	Healthcare Worker	1 SNV difference		
15	47	Family Member	1 SNV difference[§]	Same clade & lineage	> %95 very strong
	48	Healthcare Worker			
16	49	Healthcare Worker	Identical variation	Same clade & lineage	> %95 very strong
	50	Family Member			
	51	Family Member			
17	52	Healthcare Worker	Identical variation	Same clade & lineage	>%95 very strong
	53	Healthcare Worker			
	54	Healthcare Worker	>2 SNV difference		< %75 weak
18	55	Family Member	1 SNV difference	Same clade & lineage	>%95 very strong
	56	Family Member	Identical variation		
	57	Healthcare Worker	Identical variation		
19	58	Healthcare Worker	2 SNV difference	Same clade & lineage	> %95 very strong
	59	Family Member	2 SNV difference		
	60	Family Member	Identical variation		
20	61	Healthcare Worker	Identical variation	Same clade & lineage	>%95 very strong
	62	Family Member			
	63	Family Member			

ML: Maximum Likelihood, SNV: Single Nucleotide Variant, MNV: Multi-Nucleotide Variant, §: Uncertain variation due to unknown base
